# Unusual *Methylobacterium fujisawaense* Infection in a Patient with *Acute Leukaemia* Undergoing Hematopoietic Stem Cell Transplantation: First Case Report

**DOI:** 10.1155/2010/313514

**Published:** 2010-04-08

**Authors:** Rosa Fanci, Giampaolo Corti, Alessandro Bartoloni, Enrico Tortoli, Alessandro Mariottini, Patrizia Pecile

**Affiliations:** ^1^Haematology Unit, Department of Critical Care Medicine and Surgery, Careggi Hospital, University of Florence, School of Medicine, 50134 Florence, Italy; ^2^Infectious Disease Unit, Department of Critical Care Medicine and Surgery, Careggi Hospital, University of Florence, School of Medicine, 50134 Florence, Italy; ^3^Microbiology Laboratory, Azienda Ospedaliero-Universitaria Careggi, Careggi Hospital, Florence, Italy; ^4^Genetics and Citogenetics Unit, Azienda Ospedaliero-Universitaria Careggi, Careggi Hospital, Florence, Italy

## Abstract

Microorganisms of the genus *Methylobacterium* are facultative methylotrophic, gram-negative rods that are ubiquitous in nature and rarely cause human disease, mostly in subjects with preexisting causes of immune depression. *Methylobacterium fujisawaense*, first proposed as a new species in 1988, has never been reported as a bacterial agent of human infections so far. Here we describe a case of *M. fujisawaense* infection in a relapsed acute leukaemia undergoing unrelated allogeneic hematopoietic stem cell transplantation. Molecular identification of an *M. fujisawaense* strain was obtained from multiple mycobacterial blood cultures.

## 1. Introduction

The genus *Methylobacterium*, first proposed by Patt in 1976, consists of more than 20 strictly aerobic species of pink-pigmented, facultative methylotrophic, gram-negative rods that are widely distributed in nature and have been occasionally isolated from aqueous environments (as discussed by Furuhata et al. [1]). They rarely cause human disease, mostly in immunocompromised patients with solid or hematologic neoplasms, AIDS, renal failure, tuberculosis, or alcoholism (as discussed by Truant et al. [2]). Within such genus, *Methylobacterium mesophilicum *is the species more frequently reported as responsible for infections in humans (as discussed by Sanders et al. [3]). *Methylobacterium fujisawaense*, first described by Green in 1988 (as discussed by Green et al. [4]), has never been isolated from humans so far; here we report its isolation from the blood of an adult patient undergoing unrelated allogeneic hematopoietic stem cell transplantation (HSCT) after relapsing acute myeloid leukemia (AML).

## 2. Case Presentation

A 57-year-old Caucasian female was admitted to the haematology unit in May 2006 for AML, M2 FAB subtype, CD 34+, normal karyotype, and FLT3+ by internal tandem duplications. After standard induction chemotherapy and consolidation, the patient underwent a peripheral autologous HSCT while in complete remission because of unavailability of a matched-related donor. In April 2007, AML relapsed and a course of reinduction therapy was started. In June 2007, the patient underwent a new peripheral HSCT from an unrelated donor, without any serious infectious complication in the aplastic period. Antibacterial and antifungal prophylaxis consisted of oral ciprofloxacin (500 mgx2/day) and oral itraconazole (400 mg/day) was started on day −7. CMV prophylaxis was with ganciclovir 10 mg/kg from day −7 to −2 and foscarnet 90 mg/kg from day +4 to +20. GVHD prophylaxis consisted of cyclosporine/methotrexate and antithymocyte globulin (ATG).

Complete engraftment was on day +21. On day +30, while blood examination revealed a neutrophil count >500/mmc and a lymphocyte count of 1.7 × 10^9^/L, afebrile episode (39°C) with chills and malaise occurred. Blood cultures were obtained from both peripheral vein and central venous catheter (CVC, Groshong) by Bactec Plus Aerobic/F and Bactec Plus Anaerobic/F bottles. After incubation for five days and reading by the instrumentation Bactec 9240 (Becton, Dickinson and Co. Sparks, USA), blood cultures were positive for a *Pseudomonas aeruginosa *strain that was susceptible to amikacin, imipenem, meropenem, cefepime, ceftazidime, piperacillin, and piperacillin/tazobactam, so intravenous piperacillin/tazobactam 4.5 g tid was initiated. Chest X-ray and pulmonary CT scan performed after seven days of persistent fever showed a cavitary lesion interpreted as a possible mycotic infection. Though *Aspergillus* galactomannan antigenemia test and cultures from both sputum and bronchoalveolar lavage (BAL) were negative, antifungal therapy with intravenous voriconazole 200 mg bid and then with liposomal amphotericin B 3 mg/kg/day was added. After a clinical improvement, the patient was discharged with oral voriconazole treatment. In the first week of September 2007, on the occasion of a new febrile episode, a second CT scan did not reveal lung improvement. After her admission to the Infectious Diseases unit in September, six blood cultures (three from peripheral vein and three from CVC), sputum, BAL samples and *Aspergillus *galactomannan antigenemia were collected for microbiological investigations. It was not possible to obtain cultures through pulmonary biopsy.

Respiratory samples grew a strain of *P. aeruginosa *with a resistance phenotype identical to that of the previously isolated strain. A strain with a similar resistance profile was also isolated from a large necrotic mouth ulcer occurring at that time; antibiotic therapy with intravenous meropenem 1 g tid was promptly started, with transitory improvement. On September 13, local signs of insertion infection appeared, so CVC was removed and its tip was cultured, but no bacterial or fungal growth was observed. The patient continued to suffer from infrequent episodes of low-grade fever (37.5°C) for 10 days. Among the six blood samples taken in September, the ones investigated for aerobic and anaerobic bacteria remained negative. On the other part, five out of six ones aiming to 5 mycobacteria obtained by Bactec 13A bottles, incubated for eight weeks, and read by the instrumentation Bactec 460TB (Becton, Dickinson and Co. Sparks, USA), yielded a non-acid-fast bacillus. Such blood cultures were reported as contaminated but, because of its unique features, the organism was further investigated and two weeks later identified as *M. fujisawaense *by means of genetic sequencing of 16S rDNA. The genetic sequencing was carried out with universal primers (5′-GTATTACCGCGGCTGCTG-3′ and 5-AAGAGTTTGATCATGGCTCAF-3′) using BigDye Terminator chemistry and an AB 3730 DNA sequencer (Applied Biosystems).

## 3. Susceptibility

Testing performed on Muller Hinton using *E*-test method revealed susceptibility only to imipenem (MIC, 0.125 mcg/ml), so confirming the data of the medical literature(as discussed by Zaharatos et al. [5]). A new sputum sample collected after ten days of treatment grew a *P. aeruginosa *strain resistant to carbapenems but still susceptible to the other drugs tested. The intravenous therapy was then changed to cefepime 2 g bid plus amikacin 1 g once daily. On September 30, the patient, still afebrile, was discharged with the prescription of continuing the latter antimicrobial therapy for a total of four weeks. At the end of October 2007, the patient was readmitted to the haematology unit with high fever and a relapse of AML, in absence of graft-versus-host disease (GVHD) symptoms. After temporary reduction of fever, a persistent, high (40°C) temperature spike developed, and the patient died four days apart with a clinical picture of septic shock although in absence of any bacterial isolation from new blood samples. No autopsy was performed.

## 4. Discussion

Members of the genus *Methylobacterium *are ubiquitous in nature and can be detected in soil and freshwater environments including drinking water (as discussed by Gallego et al. [6]). *M. fujisawaense* was proposed as a new species in 1988 (as discussed by Green et al. [4]); so far neither it has been reported as a human pathogen nor it has been isolated from clinical specimens. In contrast, *M. mesophilicum *has been isolated from blood, bone marrow, skin, sputum, pleural effusion, and dyalisate of patients with clinically suspected infection (as discussed by Sanders et al. [3]). Scanty information concerning *M. fujisawaense *reports its isolation from tap water and its susceptibility to imipenem and tetracycline only, (as discussed by Furuhata et al. [1]) whereas most *Methylobacterium *species are sensitive even to aminoglycosides, ceftizoxime, ceftriaxone, ciprofloxacin, and cotrimoxazole (as discussed by Brown et al. [7]). Discordant carbapenem susceptibility, with sensitivity to imipenem and resistance to meropenem, seems to be a distinctive feature of *Methylobacterium *species (as discussed by Zaharatos et al. [5]). We report here, for the first time, the isolation of a *M. fujisawaense *strain from blood cultures during a febrile episode in an immunocompromised patient undergoing HSCT after relapsing AML. As our patient was previously infected by a multiresistant *P. aeruginosa *strain likely causing pulmonary infiltrates with necrotizing lesions, the role of this microorganism remains unclear. The fastidious growth of *M. fujisawaense *may well explain the lack of previous clinical reports. In this case, it was fortuitously detected in multiple mycobacterial blood cultures. Indeed, *M. fujisawaense *was able to grow in the radiometric broth specific for mycobacteria, and its detection was made possible by the longer incubation of mycobacterial blood cultures in comparison with the routine ones. Clinical interpretation of microbiological tests can be sometimes problematic, and conclusions should be drawn with prudence. However, the fact that *M. fujisawaense *was isolated from multiple blood samples, collected from both peripheral vein and CVC, suggests the presence of a systemic infection caused by this organism and excludes the hypothesis of contamination or colonization in our ward; moreover, laboratory contamination was very improbable, as blood transfer from the collection tube to the culture bottle by a sterile syringe and under laminar flow cabinet was the only operation carried out in the laboratory. After catheter removal, *M. fujisawaense *was no longer found in blood cultures, even if the patient was never treated with imipenem. Actually as CVC is the most common portal of *Methylobacterium *infection, most experts recommend catheter removal, as part of treatment along with appropriate antibiotic regimens (as discussed by Sanders et al. [3] and Fernandez et al. [8]). Although rare, infections with *Methylobacterium *species are now reported—particularly in immunocompromised patients—because of the improvement of identification methods. Isolation of those unusual pathogens should not be disregarded by microbiologists, their potentially infectious role should not be denied by clinicians, and all possible sources should be investigated. The availability of molecular tools is crucial to correctly identify rare microorganisms and contribute to the emergence of previously unrecognized diseases.
